# Acute Toxicity of TiO_2_ Nanoparticles to *Ceriodaphnia dubia* under Visible Light and Dark Conditions in a Freshwater System

**DOI:** 10.1371/journal.pone.0062970

**Published:** 2013-04-29

**Authors:** Swayamprava Dalai, Sunandan Pakrashi, Natarajan Chandrasekaran, Amitava Mukherjee

**Affiliations:** Centre for Nanobiotechnology, VIT University, Vellore, Tamil Nadu, India; Osaka University, Japan

## Abstract

The ever increasing industrial and consumer applications of titanium dioxide nanoparticles (TiO_2_ NPs) raise concern over the possible risk associated with their environmental exposure. Still, the knowledge regarding nanoparticle behavior in a freshwater ecosystem is lacking. The current study focuses on the toxicity of TiO_2_ NPs towards *Ceriodaphnia dubia* (a dominant daphnid isolated from the freshwater) under two different conditions; (1) light and dark photoperiod (16:8 h) and (2) continuous dark conditions, for a period of 48 h. An increase in toxicity was observed with an increase in the concentration, until a certain threshold level (under both photoperiod and dark conditions), and beyond which, reduction was noted. The decrease in toxicity would have resulted from the aggregation and settling of NPs, making them less bioavailable. The oxidative stress was one of the major contributors towards cytotoxicity under both photoperiod and dark conditions. The slow depuration of TiO_2_ NPs under the photoperiod conditions confirmed a higher NP bioaccumulation and thus a higher bioconcentration factor (BCF) compared to dark conditions. The transmission electron micrographs confirmed the bioaccumulation of NPs and damage of tissues in the gut lining.

## Introduction

The multitude of usage of titanium dioxide nanoparticles (TiO_2_ NPs) in various consumer and industrial products has increased the frequency of its potential environmental release [1], [2]. The estimated worldwide production of TiO_2_ NPs is 10000 tons/year for 2011–2014, and 2.5 million metric tons/year by 2025. The direct evidence of environmental release of TiO_2_ NPs from the domestic products has been documented [3].

Most of the previous studies on risk assessment and environmental impacts of TiO_2_ NPs have focused on their biological impacts and cytotoxicity. The studies of TiO_2_ NPs effect on aquatic organisms have mostly been done on water fleas (*Daphnia magna*, *Daphnia pulex*, *Ceriodaphnia dubia* etc.), and the 48 h mortality (EC50) was noted to be more than 100 mg L^–1^ [4], [5], [6]. In contrast, Zhu et al. [5] showed a comparatively lower 72 h EC50 of 1.62 mg L^–1^ using TiO_2_ NPs advocating the effect of increased exposure time on NP toxicity. However, no information was given on irradiation conditions. The previous ecotoxicity studies on aquatic organisms have emphasized the photocatalytic behavior of TiO_2_ NPs in the presence of simulated sunlight [7] or UV irradiation conditions [8], [9]. A recent study reported the absence of toxicity for anatase and rutile TiO_2_ NPs to *Daphnia similis* under both visible light and dark conditions even at 100 mg L^–1^ [6]. Since, there are evidences showing free radical generation by TiO_2_ NPs under visible light and dark conditions [10], [11], cytotoxicity under these circumstances needs to be evaluated.

Oxidative stress such as generation of reactive oxygen species (ROS) and their detrimental effects are considered as the main mechanisms for TiO_2_ NP toxicity [12], [13]. On the contrary, Frohlich et al., [14] advocated the possibility of oxidative stress independent cytotoxicity mechanisms of NPs. Fewer investigations have looked for other modes of toxicity such as attachment of NPs onto the organism surface [15], [16] and its accumulation in the digestive tract [17] leading to physical impairment of organism behavior or health.

Size is also an important factor determining TiO_2_ NP toxicity as smaller particles may ease up the penetration and thus accumulation process in tissues [18]. A few studies investigated the particle size effect on TiO_2_ NP ecotoxicity [7], [19]. The stability of NPs in the experimental matrix also determines its behavior and reactivity towards the aquatic organisms [20]. The aggregation potential of NPs may reduce its bioavailability and thus the ecotoxicity [21]. On the other hand, the settling down of NPs may adversely affect the sediment dwelling aquatic organisms [22]. Hence, considering all the experimental challenges, a timely modification of the conventional protocols is required [23].

The current study focuses on the cytotoxicity potential of TiO_2_ NPs towards a dominant freshwater crustacean, *Ceriodaphnia dubia*, in a lake water system. The studies were carried out under a 16:8 h light and dark photoperiod condition that is known to be a standard condition for daphnia culture and growth; and a continuous stretch of dark condition. The cytotoxicity potential of NPs followed a dose dependent trend at lower concentrations (<32 mg L^–1^), after which toxicity reduced with further increase in concentration, probably due to the aggregation of NPs. The oxidative stress was noted to be a major contributing factor in NP toxicity. The study also shows that, the reactivity of NPs in the experimental matrix cannot be linked with the initial NP concentration but rather with the bioavailability of NPs towards the aquatic organism. In summary, the current study emphasizes on the refinement of conventional protocols for photo-induced toxicity assessment where TiO_2_ NP reactivity under visible light and dark conditions were considered amongst the control experiments.

## Materials and Methods

### Materials

Dry titanium (IV) oxide nanopowder (TiO_2_, 99.7% Anatase, particle size: <25 nm), DCFH-DA (2′–7′–Dichlorofluorescin Diacetate) was procured from Sigma Aldrich, USA. All other chemicals were of analytical grade.

The freshwater was collected from VIT lake, Vellore, India (Vellore location: 12.93 °N 79.13 °E; elevation: 216 m; temperature range: 10°C–43°C). Since the lake is being maintained by the VIT university management, no specific permission was required for the sample collection. The study has been carried out in a laboratory away from the lake and did not involve any threat to endangered or protected species. Physical parameters of the freshwater, conductance: 4.5±0.17 mS cm^–1^; pH: 7.7±0.2; dissolved oxygen (DO): 7.4±0.49 mg L^–1^; total dissolved solids (TDS): 820±80 mg L^–1^; total organic carbon (TOC): 15 mg C L^–1^. The freshwater was filtered through Whatman no. 1 filter paper to avoid intrusion of larger colloids and then autoclaved. The filtrate was used as the test media throughout this study [24].

### Test Organism

The dominant daphnids were isolated from the lake water and were identified as *Ceriodaphnia dubia*. The daphnids were kept in a climate controlled chambers at 25±2°C on a 16:8 h (light: dark) photoperiod. Animals were fed with the green algae, *Scenedesmus obliquus*, (dominant green algae in the lake) once in two days. The culture was maintained in filtered and sterile lake water that was renewed three times a week.

### Toxicity Studies

Acute toxicity tests of TiO_2_ NPs were conducted following the modified OECD standard procedure [25]. The toxicity of TiO_2_ NPs to *C. dubia* was studied using seven different concentrations of NPs, i.e. 1, 2, 4, 8, 16, 32, 64 mg L^–1^, plus a negative control (0 mg L^–1^). Each NP concentration was interacted with ten juveniles (<24 h) of *C. dubia*, collected from a selected brood, for a period of 48 h. The daphnids were not fed during the experimental period. The experiments were carried out in two parallel sets; under photoperiod conditions (light: dark: 16: 8 h) and dark (no irradiation) conditions. Hence, to evaluate the ecotoxicity aspects of TiO_2_ NPs, the current study was carried out under light (visible) and dark photoperiod conditions and compared against continuous dark conditions. Visible light irradiation was provided by white fluorescent lamps (3000lux, 15 W, Philips, India). For dark experiments, beakers were wrapped with opaque sheets and were kept in dark chambers without intervention of any visible light. Henceforth, the light and dark photoperiod (16:8 h) conditions will be denoted as photoperiod conditions and dark (no irradiation) as dark conditions throughout the manuscript. The glass beakers were not covered fully to allow proper passage of air. Equal temperature and air flow was maintained in both the experimental setups. After the experimental period, daphnids that were not showing any sign of motion even after gentle agitation of the container were considered immobile. All experiments were conducted in triplicates. The 48 h EC50 values were calculated using a Probit method (US EPA Probit Analysis Program, Ver. 1.5).

### Oxidative Stress in *C. dubia*

The role of free radicals in causing cytotoxicity was analyzed by quantifying the generation of reactive oxygen species (ROS). The membrane permeable, non-fluorescent dye, DCFH-DA gets oxidized to fluorescent DCF in the presence of intracellular ROS and cellular esterase. Thus, the increased fluorescence intensity of DCF can be directly linked to the increased intracellular ROS generation. The ROS production was monitored in control, and NP treated animals after 48 h interaction times following the protocol described by Wang and Joseph with minor modification [26]. Five daphnids were collected after the treatment period and suspended in fresh lake water matrix. The tissues of daphnids were homogenized by ultrasonication (130 W, 1 min) and centrifuged at 7000 g for 10 min. The supernatant collected was incubated with DCFH–DA with a final concentration of 100 µM at 37°C for 30 min. The fluorescence was measured using a spectrofluorometer (SL174, ELICO) with excitation and emission wavelengths of 485 nm and 530 nm, respectively. The interference of NPs alone was subtracted from the results.

The superoxide dismutase activity (SOD) was measured following the protocol described by Wintherbourn et al. [27]. Riboflavin was used as the O_2_ generator. This method depends on the ability of the enzyme to inhibit the reduction of nitroblue tetrazolium (NBT) by superoxide generated by the reaction of photo-reduced riboflavin and oxygen.

### Total NP Uptake

To understand the effect of physical adsorption and internalization of NPs on *C. dubia* survival, total uptake analysis was done. The total uptake by daphnids included the quantification of NP attached onto the daphnids exoskeletons and its accumulation/internalization in *C. dubia* alimentary canal (and other tissues). Ten daphnids (<24 h) were exposed to the assigned concentrations of NPs for a period of 48 h. After the treatment period, 5 daphnids were collected from each exposure concentration and the organisms were washed twice in fresh sterile lake water to remove any loosely bound NPs. The washed daphnids were then subjected to acid digestion with concentrated nitric acid. The quantification of Ti metal content was done by ICP-OES analysis (Inductive Coupled Plasma-Optical Emission Spectroscopy; Perkin Elmer Optima 5300 DV, USA).

### NP Bioconcentration Kinetics in *C. dubia*

The accumulation/bioconcentration kinetics of NPs in *C. dubia* were studied, which included a 48 h uptake period followed by a 48 h depuration period. The exposure concentration was selected based on the acute toxicity results obtained that showed maximum lethality (16 mg L^–1^) under photoperiod conditions. To draw a comparison between the two experimental conditions, same NP concentration, 16 mg L^–1^ was used under dark conditions. In brief, ten juveniles of *C. dubia* (<24 h old) were exposed to 16 mg L^–1^ TiO_2_ concentration. At 0, 6, 12, 24 and 48 h of exposure (uptake times), 5 animals were collected for each concentration. The animals were washed twice in sterile lake water to remove any loosely bound NPs and then subjected to acid digestion followed by ICP–OES analysis. For depuration studies, after 48 h of exposure to TiO_2_ NPs, 5 daphnids were washed and transferred to fresh experimental matrix. After 6, 12, 24 and 48 h of depuration, Ti content in the organisms was estimated.

The TiO_2_ NPs bioconcentration factor (BCF) was calculated for *C. dubia* in photoperiod and dark conditions as the ratio of the whole body TiO_2_ NP concentration (mg kg^–1^, after the 48 h depuration process) to the actual aqueous concentration of NPs (mg L^–1^) at an initial concentration of 16 mg L^–1^ [28].

### Microscopic Studies

It was observed that the tissues of daphnids degrade rapidly as soon as they die (immobilized). Hence live organisms (mobile) were collected for microscopic studies. An exposure concentration of 16 mg L^–1^ was used for the microscopic studies. At least 20 daphnids were collected after the 48 h experimental period. Semi-thin sections (0.5–1 µm) of mid gut, stained with toluidine blue were observed under light microscope (Zeiss Axiostar Microscope, USA). Ultrathin 5–10 mid gut sections were taken to study the internalization of NPs in the alimentary canal and changes in cellular structure under transmission electron microscope (Philips CM12 Transmission Electron Microscope, Netherlands).

### Stability and Sedimentation of TiO_2_ NPs in Experimental Matrix

Dispersion of NPs (1, 16 and 64 mg L^–1^) was prepared in sterile lake water using ultrasonication (130 W, 5 min, Sonics, USA). The concentrations of particles were decided according to the cytotoxicity studies carried out. The dispersions were kept in static conditions under a photoperiod of visible light:dark::16:8 h and under continuous dark conditions. The visible light intensity was 3000 lux (TL–D Super 80 Linear fluorescent tube, Philips, India) and dark condition was maintained by keeping the sample vials in dark rooms. Equal temperature and air flow was maintained in both the experimental setups. The undisturbed top layer of NP dispersion (1 cm) was collected time to time for 48 h. The hydrodynamic particle size was immediately monitored using Dynamic Light Scattering particle size analyzer, and the respective UV–Vis spectra were collected. The experiments were carried out in triplicates to calculate the standard error. The concentration of TiO_2_ NP at the top layer of dispersion was analyzed by ICP–OES, after acid digestion of the sample.

### Statistical Analysis

The cytotoxicity experiment and biochemical assays (ROS, SOD) were repeated at least three times and the data are presented as mean ± standard error. In all experiments, NP treated samples were compared with respective controls. The statistical analysis was done using t-test at p<0.05. Graph Pad Prism (Version 6.01) was used for all the statistical analyses.

## Results and Discussion

### Toxicity Studies

**Figure 1:**
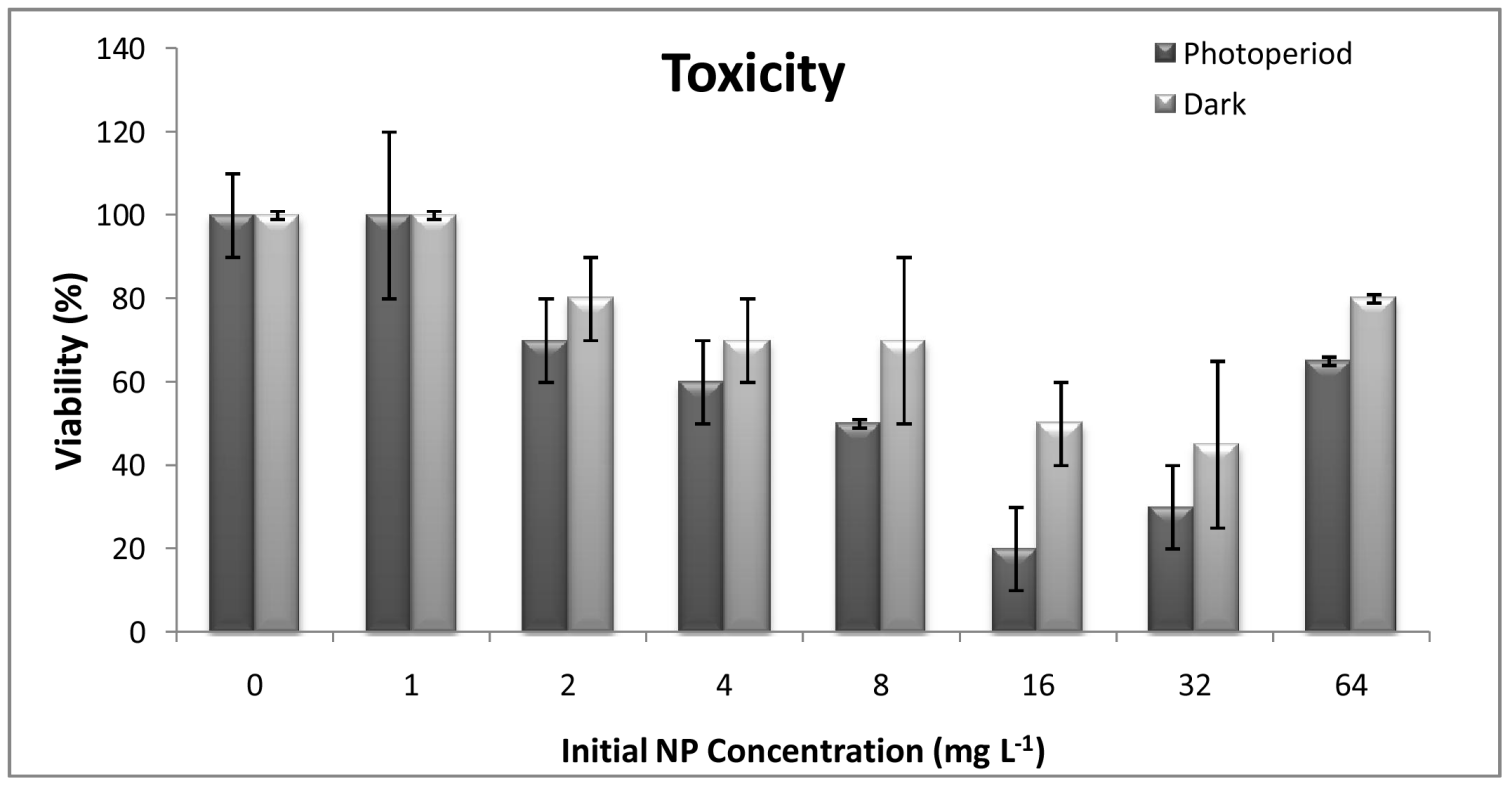
Toxicity assessment. Toxicity of TiO_2_ NPs under photoperiod and dark conditions with respect to concentrations (n = 5).

The photoperiod toxicity experiments showed a concentration dependent reduction in viability till 16 mg L^–1^ (viability 20±10%). However, at 32 mg L^–1^, a slightly higher viability of the organisms was noted (30±10%) as compared to 16 mg L^–1^ (p>0.05) which further increased till 65±10% (p<0.05) at 64 mg L^–1^ (Figure 1). The increased survival of daphnids at higher concentrations can be attributed to the reduced NP toxicity owing to its rapid aggregation at higher concentrations [21]. A 50% reduction in viable (mobile) organisms was recorded around 8 mg L^–1^ [EC50 = 8.26 mg L^–1^, photoperiod]. Under dark conditions, the maximum toxicity was observed at 32 mg L^–1^, where nearly 50% (±10%) reduction in viability was noted [EC50 = 27.45 mg L^–1^, dark]. As observed for photoperiod experiments, in dark conditions an increase in viability was observed at 64 mg L^–1^ concentrations, showing nearly 80% (±10%) survival (p>0.05).

Studies on the acute ecotoxicity of TiO_2_ NPs to daphnids are limited. Lovern and Klapper [19] reported concentration dependent mortality of *D. magna* on exposure to filtered TiO_2_ NPs (∼ 30 nm), with an LC50 5.5 mg L^–1^. An EC50 greater than 100 mg L^−1^ was reported by Warheit et al. [4] and Zhu et al. [5] for TiO_2_ (100–140 nm) for *D. magna* after 48 h of exposure. Wiench et al. [29] found EC50 more than 100 mg L^−1^ for uncoated and coated nanoparticles and uncoated non-nanoparticles of TiO_2_. In a recent study, Amiano et al. [30] showed the EC50 value of 3.4 mg L^–1^ TiO_2_ after exposure to 0.56 mW cm^–2^ UVA radiation using river water as the matrix. In another study, Marcone et al. [6] showed no toxicity of P25 Degussa in the visible photoperiod or dark conditions at the maximum concentration (100 mg L^−1^) tested. According to these studies, TiO_2_ NPs appears to exert no or low acute toxicity to eco-relevant species. The EC50 values observed under photoperiod (8.26 mg L^–1^), and dark (33.65 mg L^–1^) conditions were the lowest ever reported for 48 h exposure period, which negate the previous reports showing low or no toxicity under visible light and dark conditions. The current study is perhaps the first of its kind to report cytotoxicity of TiO_2_ NPs under dark conditions.

### Oxidative Stress Analysis

**Figure 2:**
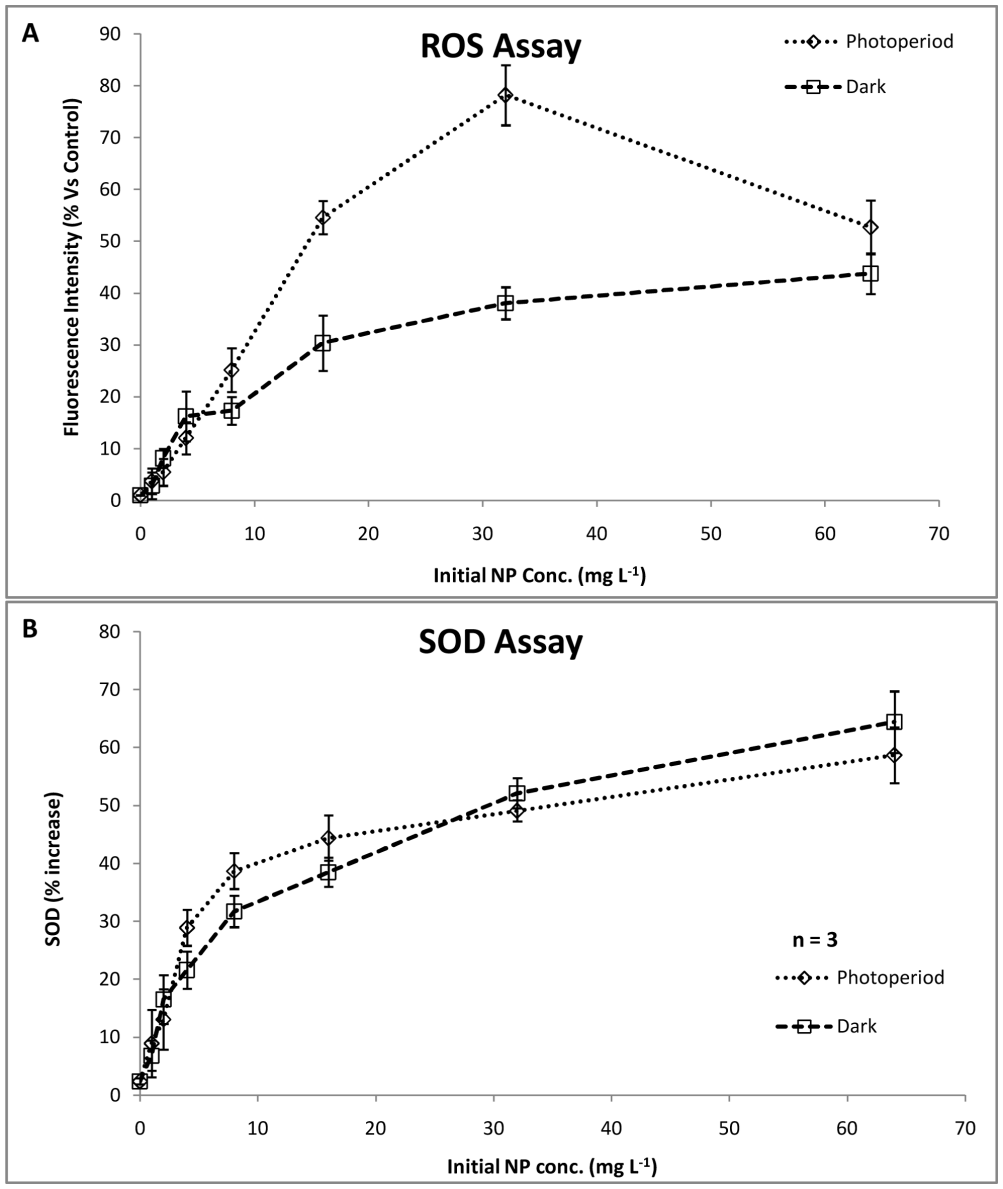
Oxidative Stress assessment in *C.* *dubia*. (A) ROS quantification under photoperiod and dark conditions showed a concentration dependent increase. Nanoparticle interaction shows greater ROS generation. (B) SOD activity increased with increasing concentration. (n = 3).

To determine the role of oxidative stress in daphnids immobilization, evaluation of reactive oxygen species (ROS) and superoxide dismutase (SOD) was done. The ROS assay results showed a concentration dependent increase in oxidative stress on the daphnids till 32 mg L^–1^ NP concentration (Figure 2A). In a recent study on marine phytoplankton, Miller et al., [9] showed an increased ROS generation with concentration under UV irradiation conditions. However, the ROS amount reduced at 64 mg L^–1^ (52.67±5.2%) was statistically significant with respect to the ROS at previous concentration of 32 mg L^–1^ (78.17±5.8%, p<0.05). The notable decrease in ROS can be attributed to the loss of reactivity of NPs probably due to aggregation. On the other hand, under dark conditions, statistically significant (p<0.05) and continuous increase in ROS generation was recorded as compared to control. The maximum ROS was noted at 64 mg L^–1^ (43.75±3.9%). The photoperiod treated samples showed a very high ROS generation as compared to dark (p<0.05), which can be attributed to the generation of free radicals under visible light conditions [11]. Fenoglio et al. [31] showed the possibility of free radical generation even under dark conditions.

The SOD assay showed a concentration dependent increase under both photoperiod and dark conditions (Figure 2B). However, at higher concentrations (≥32 mg L^–1^), SOD in the dark was found to be more (5.21±0.3%, 6.44±0.85%) as compared to photoperiod (4.91±0.41%, 5.87±0.62%). This suggested higher resistance of the organisms under dark with respect to photoperiod [32], [33]. These observations corroborated with the toxicity results.

Although, the oxidative stress induced by NPs appeared to the major causative factor, the contribution of other factors cannot be overlooked. Frohlich et al. [14] suggested the possibility of oxidative stress independent cytotoxicity mechanisms of NPs.

### Total NP Uptake and Bioconcentration

**Figure 3:**
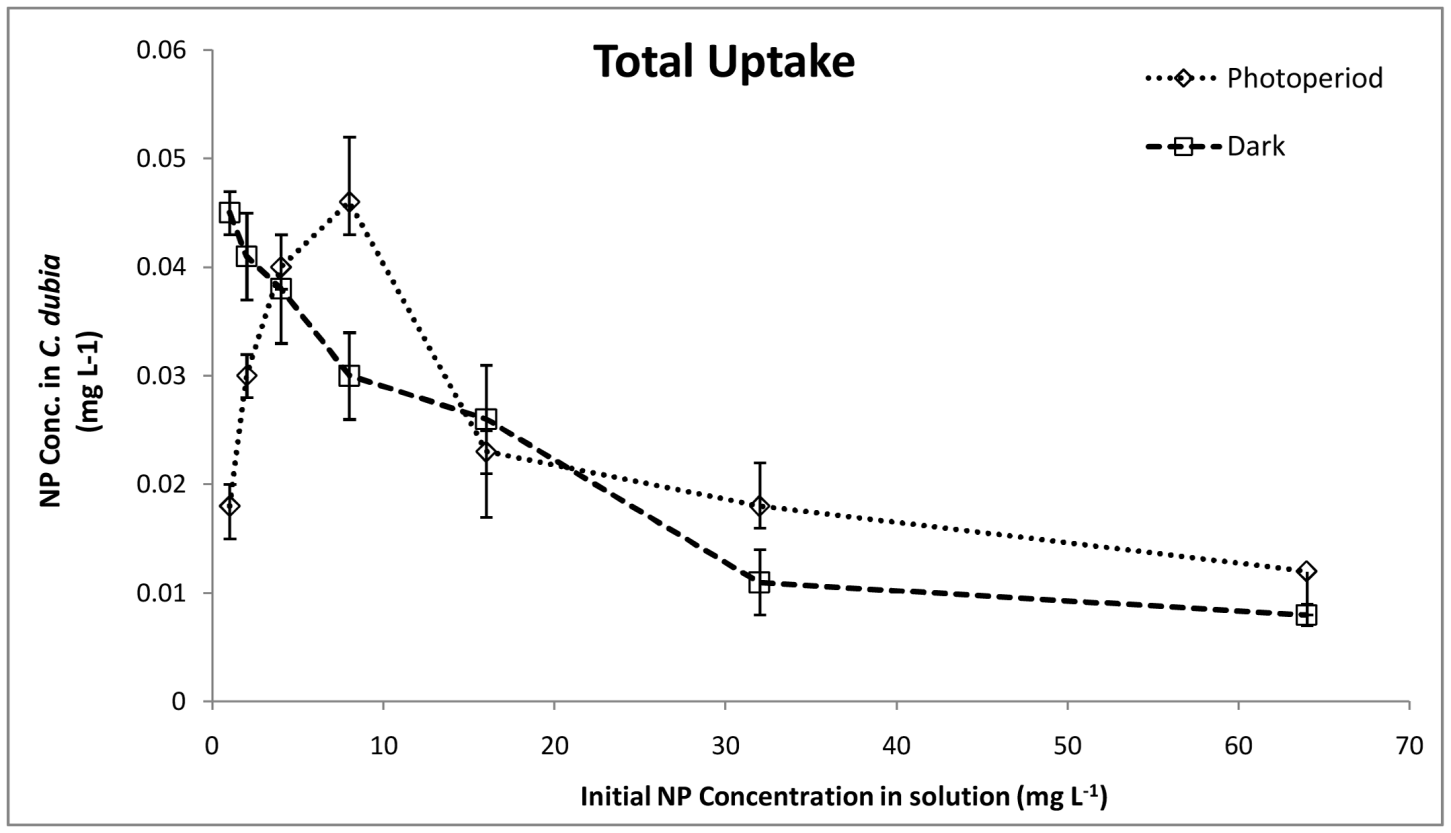
Total Uptake. Quantification of total NP uptake (ICP-OES) with increase in concentration (n = 3).

The total NP uptake by *C. dubia* (Figure 3) under photoperiod conditions increased with concentrations till 8 mg L^–1^ (0.046±0.003 mg L^–1^), after which, a constant decrease was observed. Comparing the uptake data with toxicity results for photoperiod reactions, it was inferred that the total uptake of NPs may contribute to the toxicity observed till 8 mg L^–1^. A reduced total uptake at 32 and 64 mg L^–1^ can also be related to the reduced toxicity observed. These observations suggested that the aggregation at increased concentrations might affect NP uptake or sorption by *C. dubia*. Notably, at 16 mg L^–1^ a decrease in NP total uptake did not correlate with the observed increase in toxicity (Figure 1). This confirmed the contribution of additional factors such as oxidative stress, and the possible physical damage caused by NPs. Under the dark conditions, the uptake value was highest at the lowest concentrations (0.045±0.002 mg L^–1^ at 1 mg L^–1^) which gradually decreased with increase in NP concentration. No definite correlation was observed between increases in toxicity and decreased total uptake under the dark conditions which suggested contribution of other physicochemical factors. Moreover, any disturbance in the normal molting pattern of daphnids can also influence the total NP uptake values [21]. Comparing the uptake values in photoperiod and dark conditions, it can be deduced that the daphnids have undergone a normal molting under dark conditions and till 8 mg L^–1^ in photoperiod conditions. The molting inhibition might have taken place at higher concentrations in photoperiod experiments.

**Figure 4:**
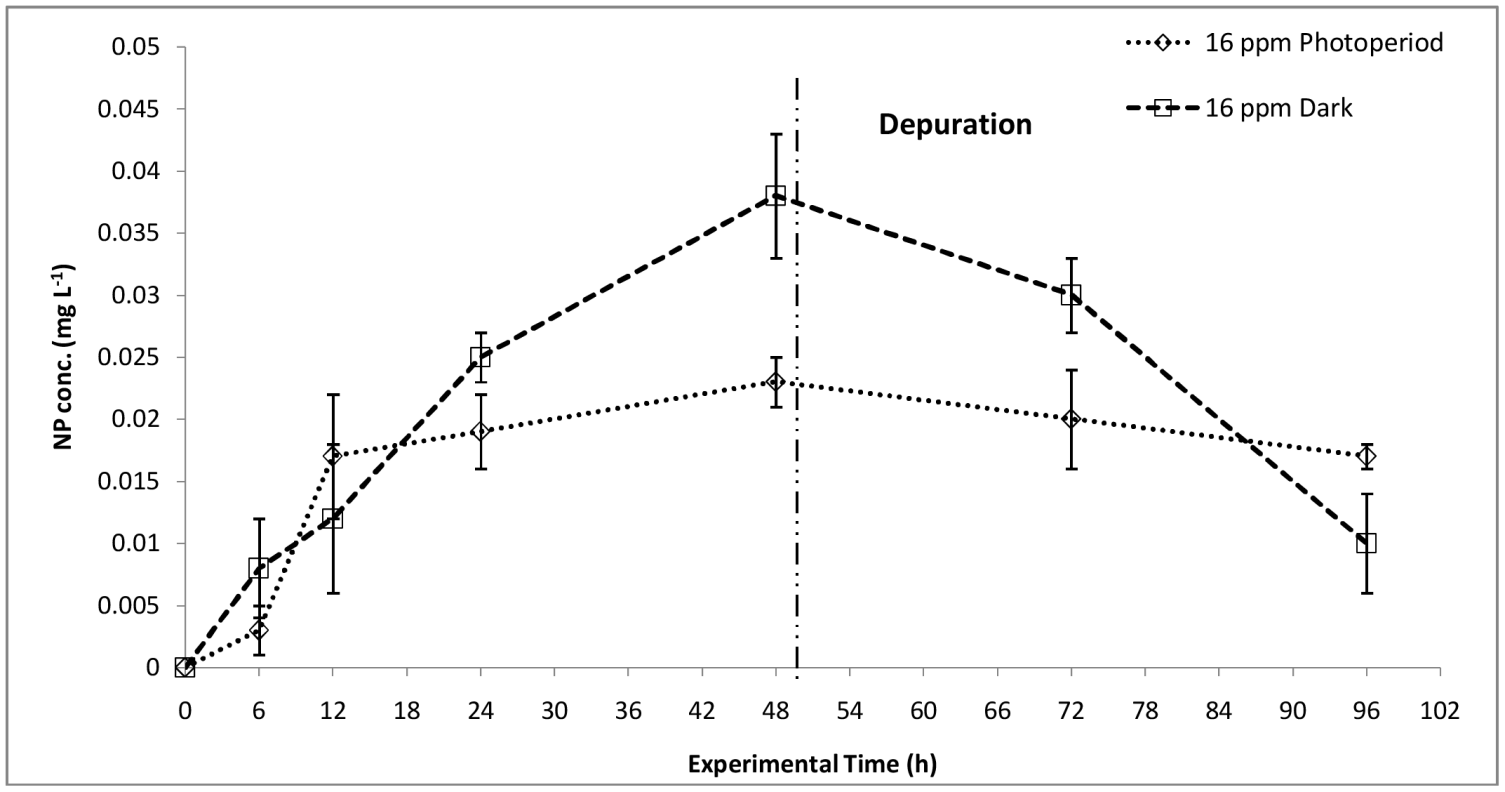
Bioaccumulation. NP uptake (48 h) and depuration (48h) kinetics under photoperiod and dark conditions at 16 mg L^–1^ NP concentration (n = 3).

Since, the total uptake includes internalization as well as attachment of NPs to the exoskeletons of daphnids, depuration of NPs was studied, and bioconcentration of NPs in the organisms was calculated with respect to time (Figure 4). The bioconcentration of NPs was quantified at 16 mg L^–1^ exposure concentration, which showed maximum lethality. The bioconcentration kinetics under photoperiod conditions showed a steady increase in NP concentration with time, which went till 0.023±0.006 mg L^–1^ at 48 h. The depuration rate did not show a sharp decrease, suggesting accumulation of NPs (0.017±0.001 mg L^–1^) in the daphnids’ alimentary canal. Under dark conditions, however, the depuration rate was faster and the final NP concentration (after 48 h) was noted to be 0.01±0.004 mg L^–1^. This showed lesser accumulation of NPs in the organisms’ alimentary canal and other tissues.

The bioconcentration factor (BCF) was calculated to be 502.13 in photoperiod and 318.05 in dark conditions for 16 mg L^–1^ concentration (Table 1). The lesser NP bioconcentration in dark treated samples can be connected to the lesser toxicity observed and vice versa. The localization of TiO_2_ NPs in the digestive tract of *D. magna*, displaying difficulty in eliminating the NPs from the body and thus showing an increased bioconcentration factor (BCF) was reported by Zhu et al. and Pinheiro et al. [5], [17]. Pinheiro et al. [17] have shown localization of Ti in the alimentary canal through nuclear microscopy.

**Table 1:** Bioconcentration factor (BCF) in *C. dubia* after 48 h NP treatment (n = 5).

Conc. (mg L^–1^)	Actual Conc. in Experimental matrix (mg L^–1^)	Whole body concentration (g Kg^–1^, dry weight)	BCF
16	14.72	7.39±0.38	502.13
16	13.67	4.34±0.16	318.05

### Effect on Alimentary Canal

**Figure 5:**
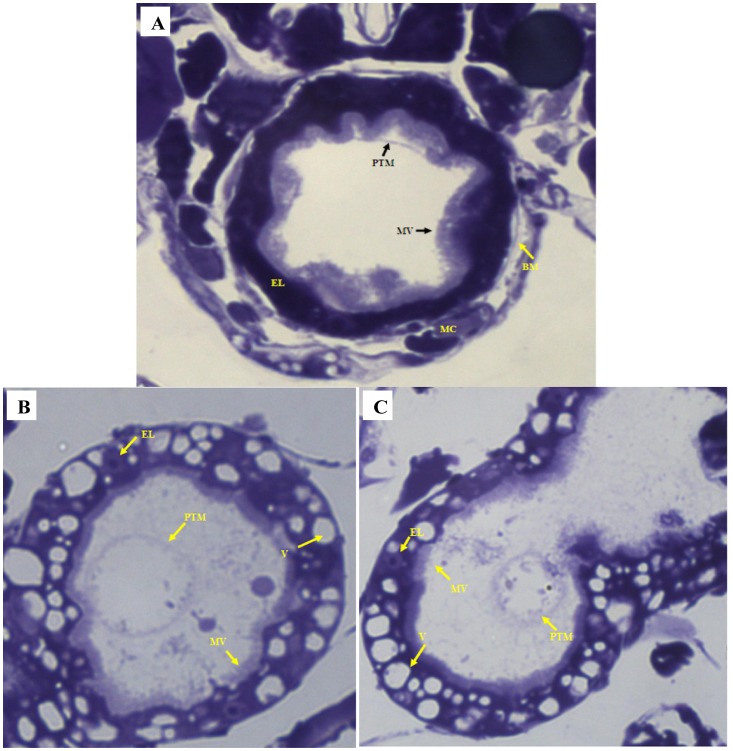
Optical microscopy of alimentary canal. (A) In the control sample, an intact gut epithelial lining with healthy cells and dense microvilli is observed. The basal membrane is intact and supported by muscle cells. The peritrophic membrane is close to the gut lining. (B) (C) In treated sample, the epithelial lining is damaged and with very few numbers of epithelial cells. A large number of vacuole formation is noted. Constriction of PTM is observed. The microvilli are few and irregular. Epithelial lining (EL); microvilli (MV); basal membrane (BM); muscle cells (MC); peritrophic membrane (PTM); vacuole (V). The gut lining of at least five different animals were observed to draw any conclusion (n = 5). Magnifications 1000X.

**Figure 6:**
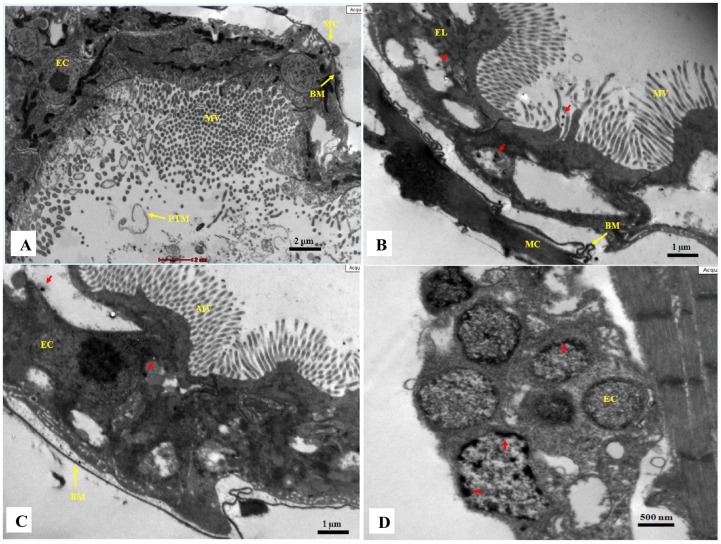
Transmission electron microscopy of the alimentary canal. (A) In control sample, an intact gut lining with healthy epithelial cells and dense microvilli is seen. Intact basal membrane is supported with muscle cells. (B) A destroyed gut lining can be seen in treated sample (photoperiod). No intact cells are visible in epithelial lining and numerous vacuole formation can be seen. The microvilli are not of uniform size. The basal membrane is irregular but supported by muscle cells. (C) In treated sample (dark), the microvilli are of uniform size and dense compared to treated samples under photoperiod. The basal membrane is irregular and the muscle cells are absent. Deposition of NPs (red arrow) can be observed in both the treated samples. (D) The epithelial cells in treated sample are seen to have an altered cytoplasm. The nucleus is either absent or deformed. Deposition of nano-sized particles (red arrow) is observed inside the cells. Epithelial lining (EL); epithelial cells (EC); microvilli (MV); basal membrane (BM); muscle cells (MC). The gut lining of at least five different animals were observed to draw a conclusion (n = 5).

The representative images of the *C. dubia* gut epithelium, after NP treatment, were shown in Figure 5 and 6. The morphological alterations in the gut lining were studied under the optical microscope. The image (Figure 5A) showed an intact alimentary canal in control cells with healthy epithelial lining and dense regular microvilli. An intact basal membrane supported by healthy muscle cells was also observed. In NP interacted daphnids, under photoperiod conditions (Figure 5B), an apparently disrupted digestive tract was observed. The epithelial lining was observed to be either deformed or cells without an intact nucleus. The formation of numerous vacuoles was also observed on the gut lining. The microvilli were not dense and highly irregular in shape and diameter. In dark treated daphnids (Figure 5C), the microvilli lining in the digestive tract were almost regular but not as dense as compared to control cells. The vacuole formation and the absence of intact epithelial cells on the gut lining confirmed the detrimental effect of NPs. The peritrophic membrane (PTM) was seen to condense and get detached from the gut lining in both photoperiod and dark treated samples (additional figures, Figure S1).

The transmission electron micrographs of control daphnids showed an intact gut epithelial lining with compact cellular arrangements and dense microvilli. The spaces between cells were tight with no significant dilatation. The basal membrane was intact and well supported by muscle cells (Figure 6A). After 48 h of 16 mg L^–1^ NP interaction, under photoperiod conditions, the epithelial cells were completely disrupted with no nucleus. The microvilli were appeared to be irregular in shape. Quite a lot hollow spaces (probably vacuoles) were observed in the gut lining confirming the destructive nature of NPs. The nano-sized particles were seen to get accumulated in the cells lining the digestive tract. Though, the basal membrane was seen to be irregular, it was well supported by muscle cells (Figure 6 B). Under dark conditions, though a lesser disturbance was observed (compared to daphnids under photoperiod conditions), yet most of the cells were dead with no intact nucleus. The muscle cells attaching to the basal membrane were not observed. The NP depositions were also observed in dark treated cells (Figure 6C), which confirmed the NP bioconcentration data. The dietary TiO_2_ NPs were found to penetrate in daphnia tissues and accumulate in the gut [17]. The epithelial cells lining the digestive tract were observed to be completely degraded or without an intact nucleus. The deposition of nano-sized particles was also observed (Figure 6D). Similar observations were reported by Heinlann et al. [34] using CuO NPs and Mendonca et al. [35] using diamond NPs to *D. magna*.

### Stability and Sedimentation of TiO_2_ NPs in Experimental Matrix

The hydrodynamic size distribution of TiO_2_ NPs (1, 16 and 64 mg L^–1^) at the undisturbed top layer was estimated by DLS analysis. For the concentration, 1 mg L^–1^, uniform distribution of particles was observed (size range of 0–500 nm) under both photoperiod and dark conditions. The z-average size was noted to be 248.15 nm under photoperiod and 293.15 nm under dark conditions. Similarly for concentrations 16 and 64 mg L^–1^, the z-average size was noted to be 517.34, 697.34 nm and 925.95, 1090.76 nm respectively under photoperiod and dark conditions (Figure S2). The above observations confirmed stable NP suspension in lake water matrix till 48 h for 1 mg L^–1^, whereas, at higher concentrations aggregation of NPs was noted. Interestingly, faster aggregation of particles was noted under dark as compared to visible light exposure conditions.

**Figure 7:**
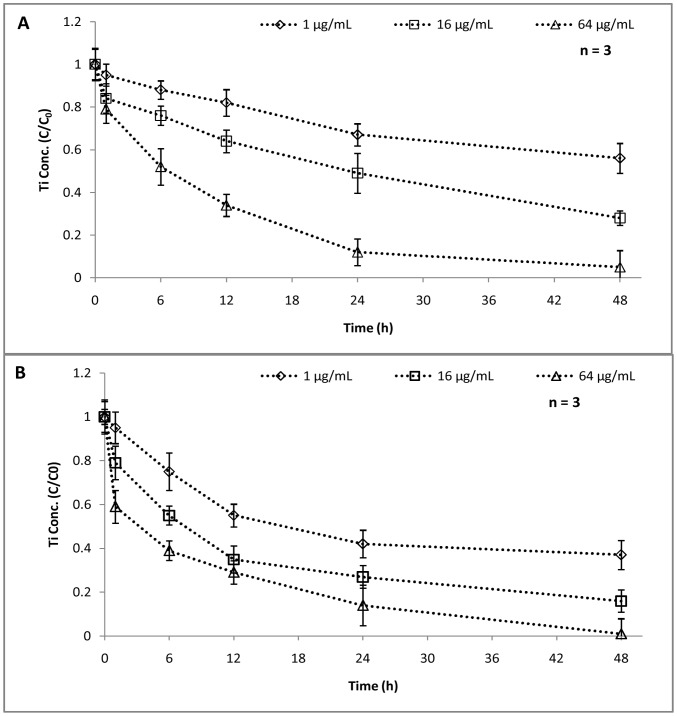
Concentration (C/C_0_) at the top layer. The concentration of NPs at the top layer (1 cm) of 1, 16, 64 mg L^–1^ NP dispersion under photoperiod conditions (A) and dark conditions (B).

**Figure 8:**
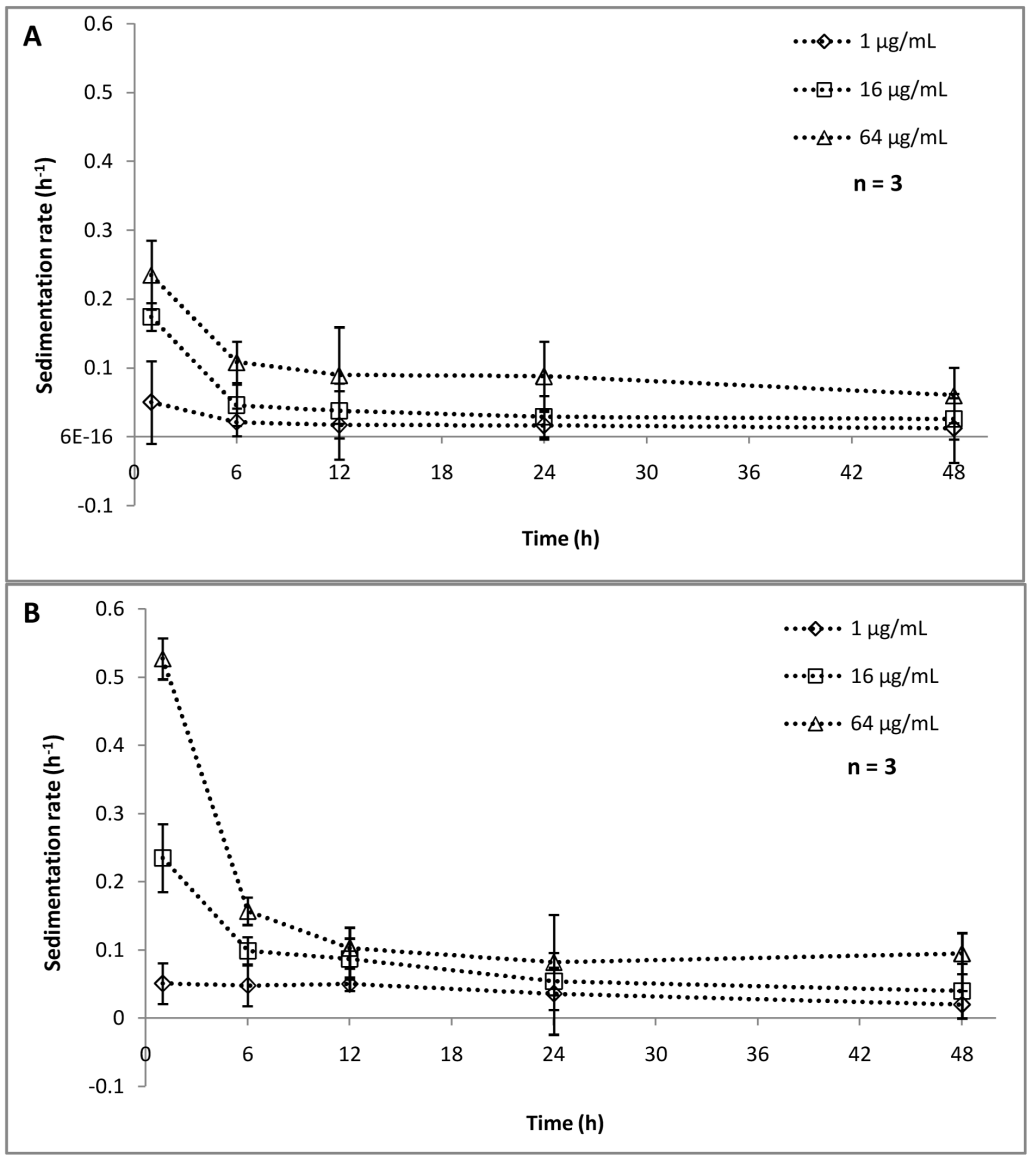
Aggregation kinetics of Nanoparticles. Aggregation kinetics of NPs in the experimental matrix under photoperiod conditions (A) and dark conditions (B). The concentrations used are 1, 16, 64 mg L^–1.^

The NP stability and sedimentation can directly be confirmed by quantifying the available concentration of NPs at the upper layer with respect to time (Figure 7). The available concentration (C/C_0_) of NPs was observed to reduce with time and with concentration. For 1 and 16 mg L^–1^ initial concentrations, in photoperiod conditions, the NP availability was high (C/C_0_>0.8) till 12 h, after which, a reduction was noted suggesting aggregation and settling of NPs (Figure 7A). On the other hand, for 64 mg L^–1^, a reduction in available concentration was observed within 6 h. A rapid settling of NPs was noted under dark conditions too (Figure 7B). The 1 mg L^–1^ NP dispersion was found to show reduced availability just after 6 h (C/C_0_ = 0.75) whereas, in 16 and 64 mg L^–1^ dispersions, the available concentration came down to 0.4 within 12 h. A steep decrease in NP concentration at the upper layer was evident in dark conditions as compared to photoperiod. The UV–Vis absorbance data (Figure S3) corroborated with the above findings. The concentration of TiO_2_ NPs is proportional to its absorbance at 330 nm, which is in accordance to the Beer–Lambert law. The aggregation kinetic rates (k_p_) were, therefore determined from the UV absorbance using the expression:
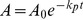
(1)Where A is UV absorbance at 330 nm at a given time t, and A_0_ is the UV absorbance at t = 0. The rate of aggregation/settling of NPs (Figure 8) showed a similar trend as that of NP concentration in the upper layer. At lower concentration (1 mg L^–1^), the rate of aggregation is quite stable and the k_p_ value ranges from 0.05 to 0.012 h^–1^ in photoperiod experiments (Figure 8A) and 0.051 to 0.02 h^–1^ in dark conditions respectively (Figure 8B). A faster aggregation was observed when concentration was increased to 16 and 64 mg L^–1^. The sedimentation rate showed that, within 6 h maximum sedimentation is happening, which was followed by almost stable settling in both conditions. As indicated earlier, the settling rate was faster in dark conditions as compared to photoperiod conditions. For 64 mg L^–1^, a sharp decrease in k_p_ value was noted (0.527 to 0.16) within 6 h of dark treatment. These observations were in corroboration with the hydrodynamic size data as mentioned earlier. The reduced bioavailability of NPs with respect to time and concentration can be linked to the cytotoxic behavior or NPs showing reduced toxicity at higher concentrations (Figure 1). In a recent study, Bennett et al. [36] reported a partial disaggregation of NPs under ambient light conditions enhancing its penetration potential as well as toxicity, which eventually supports our findings. The interrelation of NP aggregation and their toxicity aspects was well documented in several prior studies [37], [38], [39]. The accumulation of TiO_2_ NPs at the bottom of the test vessel may become a threat to the sediment dwelling organism e.g. benthic amphipod [22]. Nowack et al. [40] suggested that the nanoparticles released into the environment may not remain bare, rather may become either matrix-bound or functionalized, thus altering their cytotoxic behavior.

Connecting the stability results with the toxicity trend observed it can be assumed that the aggregation of NPs might have led to its reduced cytotoxicity at higher concentrations in both photoperiod and dark conditions. On the other hand, though aggregation may limit mobility of NPs in the environment, it may facilitate ingestion or adhesion to aquatic organism. Higher total uptake of NPs may not be the direct causative factor for organism death as observed in dark conditions. On the contrary, NP internalization and accumulation in the body (bioaccumulation) may be directly related to cytotoxicity. It should be noted that the short term exposure of *C. dubia* to TiO_2_ NPs may have serious toxicological effects because these NPs are not completely getting depurated in 48 h. Also, even if low concentrations of TiO_2_ NPs are adsorbed on the exoskeletons, adhesion of nanoparticles could interfere with the organism behavior.

## Summary

Though, several prior studies reported NP cytotoxicity towards the daphnids, there was a lack of understanding regarding NP reactivity under visible light and dark conditions. The current work is perhaps the first systematic study to unveil the possible reactivity of NPs in the photoperiod as well as dark conditions. The TiO_2_ NP behavior in different irradiation conditions may not follow a general rule which emphasizes extensive understanding. The NP stability and aggregation tendency in the environmental matrix is an important factor to be throughly studied before analyzing its toxicity towards aquatic organisms. The available concentration of NPs (bioavailability) and the bioaccumulation factor are the major parameters for assessing the ecotoxicity of TiO_2_ NPs. Although aggregation may limit mobility of NPs in the environment, it may facilitate ingestion or adhesion to aquatic organism. Also, even if low concentrations of TiO_2_ NPs are adsorbed on the exoskeletons, adhesion of nanoparticles could interfere with the organism behavior, and should therefore, be the subject of further research. Hence, the conventional protocols designed for studying NP toxicity to aquatic organisms should be considered for modification.

## Supporting Information

Figure S1 **Light microscopy of** ***C. dubia*** **alimentary canal.** (A) Control sample showing intact gut lining. EL: epithelial lining; MV: microvilli. (B) Treated sample under photoperiod conditions showing destroyed gut lining. EL: epithelial lining; MV: microvilli; V: vacuole formation. (C) Treated sample under dark conditions showing destroyed gut lining. EL: epithelial lining; MV: microvilli; V: vacuole formation; PTM: peritrophic membrane.
(TIF)

Figure S2 **DLS particle size distribution of TiO_2_ Nanoparticles.** (A) Under light conditions, the *z*-average size was noted to be 248.15, 517.34, 697.34 nm respectively. (B) Under dark condition, for 1, 16 and 64 mg L^−1^, the z- average size was 293.15, 925.95, and 1090.76 nm respectively.
(TIF)

Figure S3 **Conc. of NPs at the top layer, measured as UV-Vis absorbance.** Nanoparticle concentration was measured under photoperiod conditions (A) and under dark conditions (B) as the UV-Vis absorbance. (n = 3).
(TIF)
