# Supplementary figures and images for: Acute Toxicity of TiO2 Nanoparticles to Ceriodaphnia dubia under Visible Light and Dark Conditions in a Freshwater System
Source: PLoS One. 2013 Apr 29;8(4):e62970. doi: 10.1371/journal.pone.0062970 (PMC3639208; doi:10.1371/journal.pone.0062970)

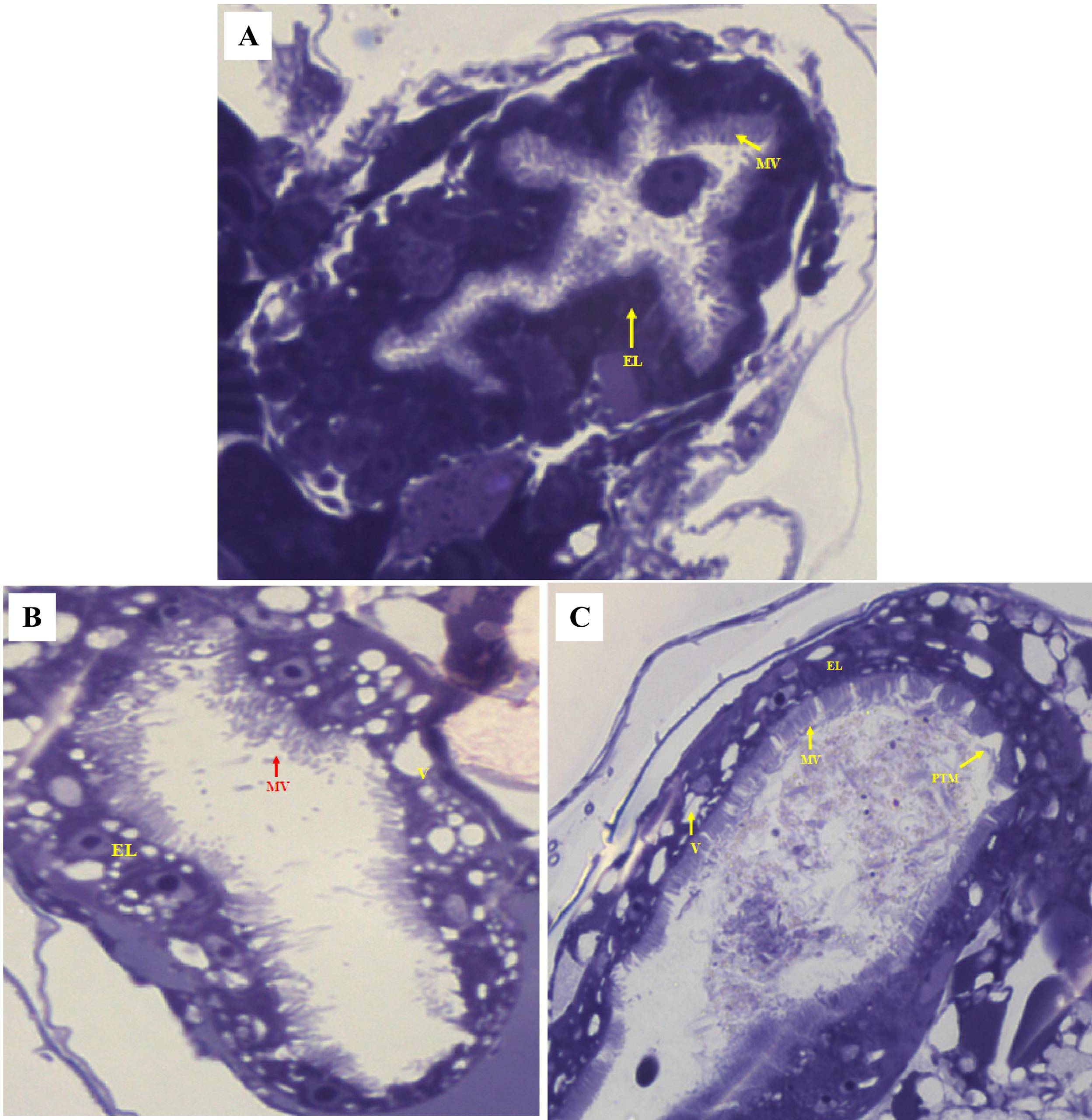

Supplement: Figure S1 — Light microscopy of C. dubia alimentary canal. (A) Control sample showing intact gut lining. EL: epithelial lining; MV: microvilli. (B) Treated sample under photoperiod conditions showing destroyed gut lining. EL: epithelial lining; MV: microvilli; V: vacuole formation. (C) Treated sample under dark conditions showing destroyed gut lining. EL: epithelial lining; MV: microvilli; V: vacuole formation; PTM: peritrophic membrane. (TIF) [file pone.0062970.s001.tif]

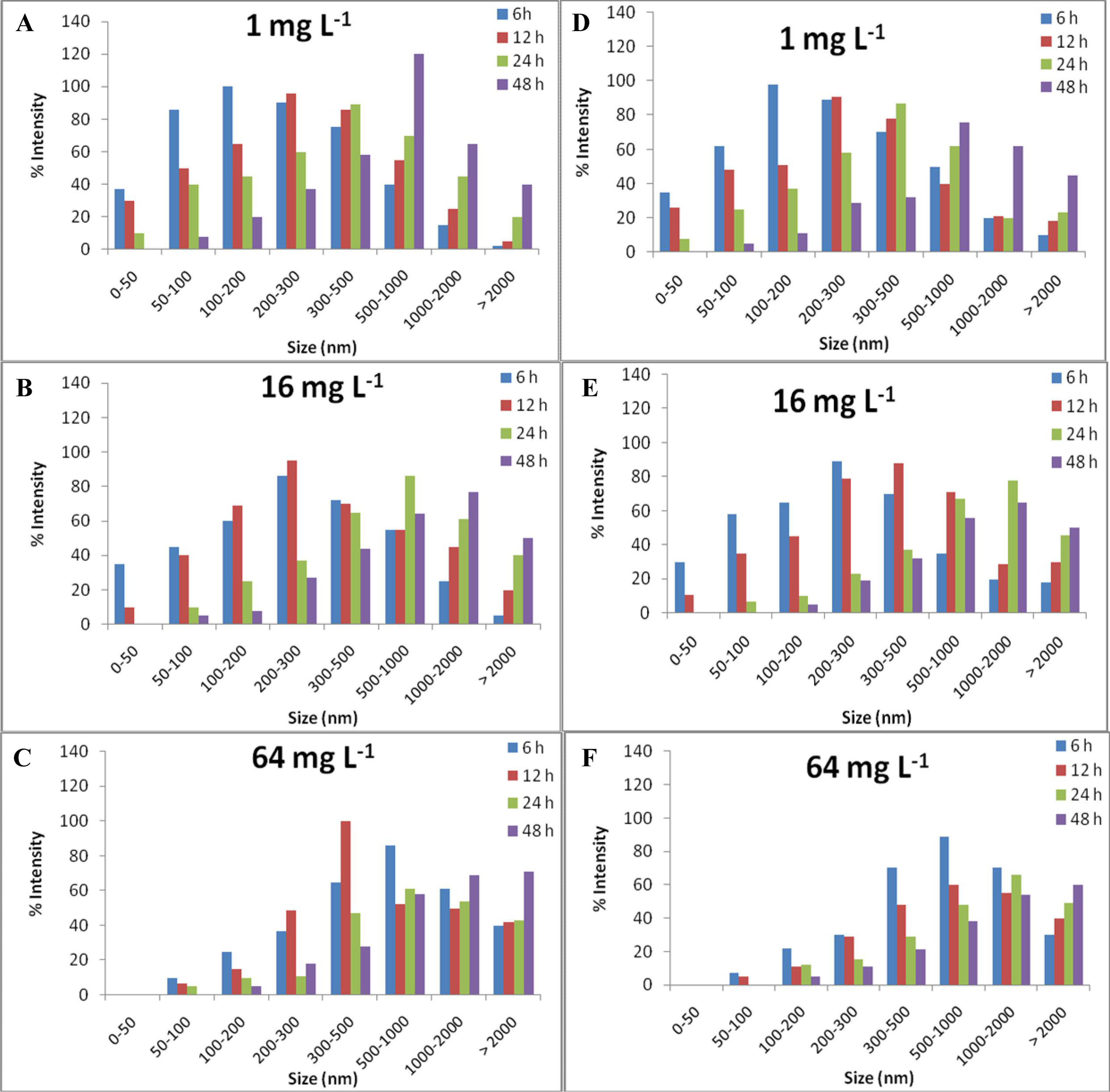

Supplement: Figure S2 — DLS particle size distribution of TiO2 Nanoparticles. (A) Under light conditions, the z-average size was noted to be 248.15, 517.34, 697.34 nm respectively. (B) Under dark condition, for 1, 16 and 64 mg L−1, the z- average size was 293.15, 925.95, and 1090.76 nm respectively. (TIF) [file pone.0062970.s002.tif]

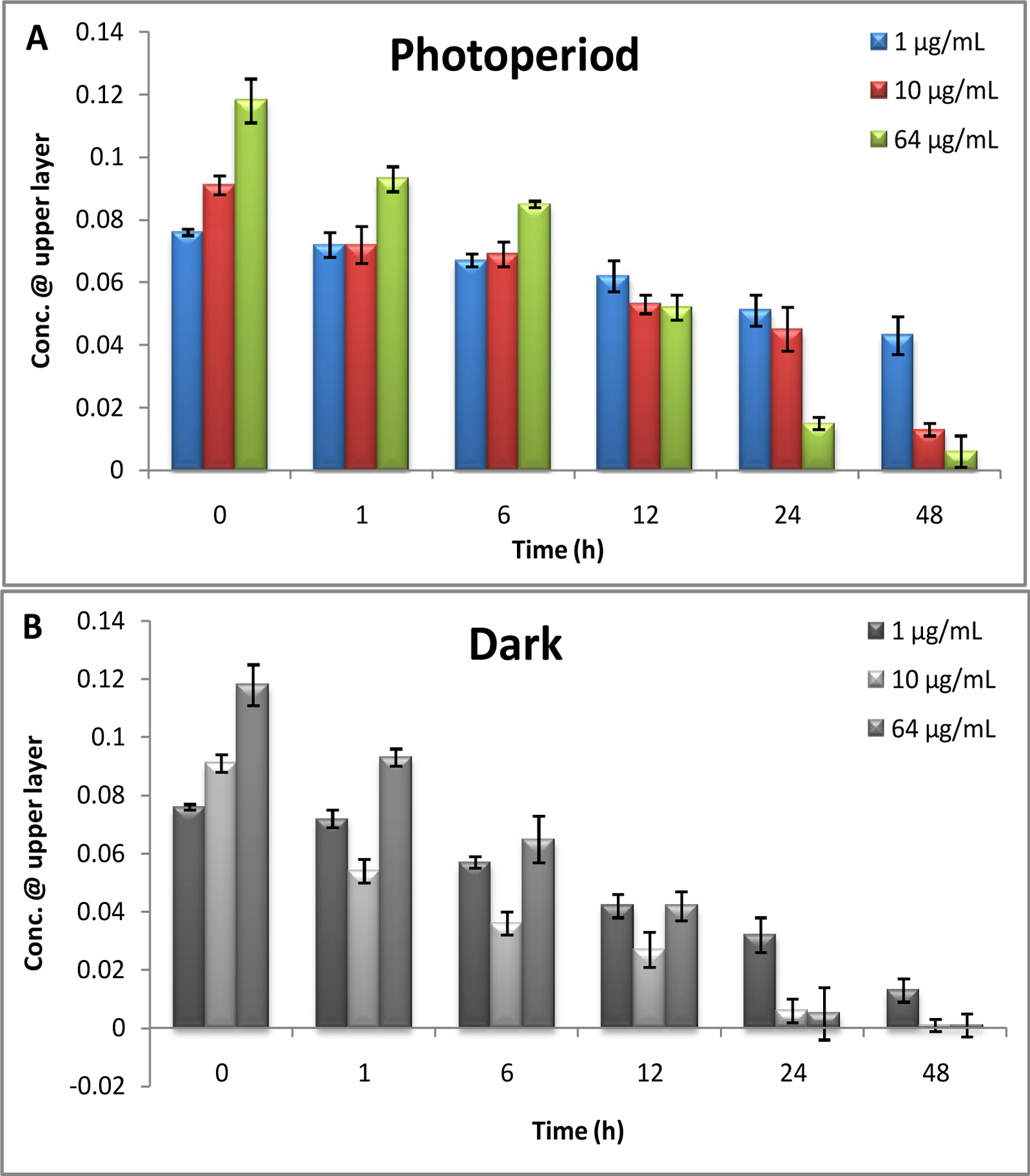

Supplement: Figure S3 — Conc. of NPs at the top layer, measured as UV-Vis absorbance. Nanoparticle concentration was measured under photoperiod conditions (A) and under dark conditions (B) as the UV-Vis absorbance. (n = 3). (TIF) [file pone.0062970.s003.tif]
